# Supplementary material for: Two is better than one: Using a single emotion lexicon can lead to unreliable conclusions
Source: PLoS One. 2022 Oct 14;17(10):e0275910. doi: 10.1371/journal.pone.0275910 (PMC9565755; doi:10.1371/journal.pone.0275910)
Supplement: S1 File — (DOCX) [file pone.0275910.s001.docx]

# **Supporting information**


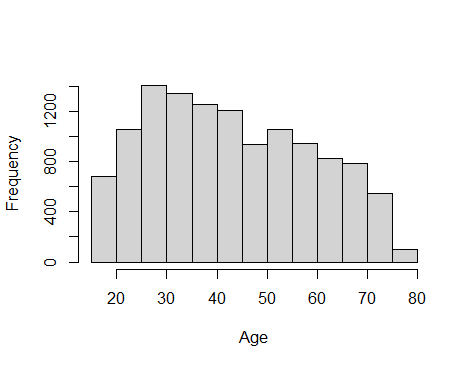


**S1 Fig.** **Distribution of age in the sample (per document).**

## **Details of the statistical models**

**S1 Table. Comparison of the model fit for the LIWC and NRC (full lexicon).**

| Affect | Lexicon | Model | *df* | AIC | BIC | log-  Likelihood | Likelihood  ratio | *p* |
| --- | --- | --- | --- | --- | --- | --- | --- | --- |
| Overall | LIWC | Linear | 5 | 49844 | 49881 | -24917 |  |  |
|  |  | Quadratic | 6 | 49741 | 49785 | -24865 | 105.45 | <.001 |
|  | NRC | Linear | 5 | 47913 | 47950 | -23951 |  |  |
|  |  | Quadratic | 6 | 47836 | 47880 | -23912 | 78.70 | <.001 |
|  | NRC | Linear | 5 | 48726 | 48763 | -24358 |  |  |
|  | (no polit.) | Quadratic | 6 | 48657 | 48702 | -24323 | 70.63 | <.001 |
|  |  |  |  |  |  |  |  |  |
| Positive | LIWC | Linear | 5 | 44534 | 44571 | -22262 |  |  |
|  |  | Quadratic | 6 | 44487 | 44531 | -22237 | 49.74 | <.001 |
|  | NRC | Linear | 5 | 40749 | 40785 | -20369 |  |  |
|  |  | Quadratic | 6 | 40709 | 40753 | -20349 | 41.43 | <.001 |
|  | NRC | Linear | 5 | 40574 | 40611 | -20282 |  |  |
|  | (no polit.) | Quadratic | 6 | 40529 | 40574 | -20259 | 46.69 | <.001 |
|  |  |  |  |  |  |  |  |  |
| Negative | LIWC | Linear | 5 | 29168 | 29205 | -14579 |  |  |
|  |  | Quadratic | 6 | 29040 | 29085 | -14514 | 129.84 | <.001 |
|  | NRC | Linear | 5 | 32520 | 32557 | -16255 |  |  |
|  |  | Quadratic | 6 | 32455 | 32499 | -16222 | 67.48 | <.001 |
|  | NRC | Linear | 5 | 31856 | 31893 | -15923 |  |  |
|  | (no polit.) | Quadratic | 6 | 31792 | 31836 | -15890 | 66.45 | <.001 |

**S2 Table. Summary of the models of emotional expression across age.** Table presents the regression coefficients with standard errors in parentheses. The unit of age is 10 years. “NRC no polit.” Refers to models based on NRC lexicon with political words removed. ^***^p < 0.001; ^**^p < 0.01; ^*^p < 0.05

|  | **Overall affect** | | | **Positive affect** | | | **Negative affect** | | |
| --- | --- | --- | --- | --- | --- | --- | --- | --- | --- |
| **Coefficients** | **LIWC** | **NRC** | **NRC no polit.** | **LIWC** | **NRC** | **NRC no polit.** | **LIWC** | **NRC** | **NRC no polit.** |
| Intercept | 3.65^***^ | 2.74^***^ | 2.06^***^ | 5.88^***^ | 5.97^***^ | 4.99^***^ | 2.20^***^ | 3.20^***^ | 2.90^***^ |
|  | (0.06) | (0.05) | (0.06) | (0.05) | (0.03) | (0.04) | (0.03) | (0.03) | (0.03) |
| Age (linear) | 0.13^***^ | 0.17^***^ | 0.08^***^ | 0.03 | 0.21^***^ | 0.10^***^ | -0.10^***^ | 0.01 | 0.00 |
|  | (0.03) | (0.02) | (0.02) | (0.02) | (0.01) | (0.02) | (0.01) | (0.01) | (0.01) |
| Age (quadratic) | -0.17^***^ | -0.12^***^ | -0.13^***^ | -0.09^***^ | -0.06^***^ | -0.07^***^ | 0.08^***^ | 0.06^***^ | 0.06^***^ |
|  | (0.02) | (0.01) | (0.02) | (0.01) | (0.01) | (0.01) | (0.01) | (0.01) | (0.01) |
| Num. obs. | 12023 | 12040 | 12042 | 11993 | 12011 | 12000 | 12056 | 12062 | 12059 |
| Residual variance | 1.66 | 1.61 | 1.62 | 1.35 | 1.16 | 1.21 | 0.72 | 0.86 | 0.82 |
| Random intercept  variance | 2.09 | 1.49 | 1.81 | 1.63 | 1.00 | 1.03 | 0.78 | 0.75 | 0.80 |
| rho | 0.56 | 0.56 | 0.58 | 0.54 | 0.45 | 0.54 | 0.56 | 0.57 | 0.55 |
|  | | | | | | | | | |

Details of the political words analysis

**
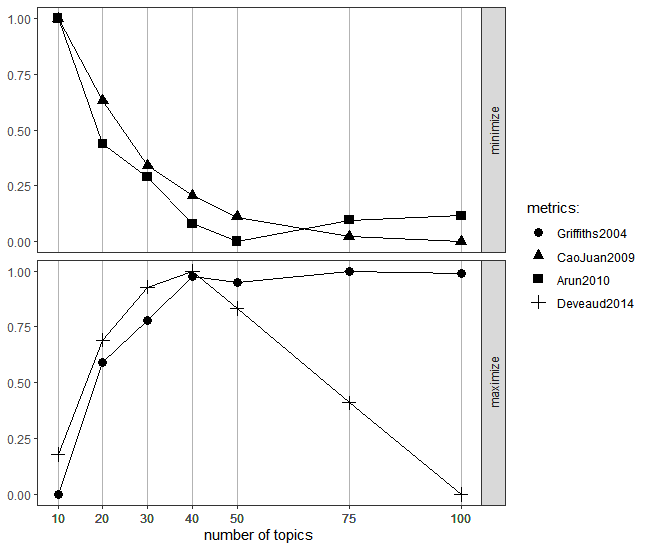
**

**S2 Fig. Comparison of LDA models with between 10 to 100 topics.**

**S3 Table. Summary of the model for age being predicted by the LDA topics.** Standard errors of the effects are presented in parentheses. ^***^p < 0.001, ^**^p < 0.01, ^*^p < 0.05

|  | **Predicting age from topics** | | | | |
| --- | --- | --- | --- | --- | --- |
| (Intercept) | 43.645^***^ | Topic 26 | -0.365^***^ | Topic 40 | 0.698^***^ |
|  | (0.265) |  | (0.055) |  | (0.052) |
| Topic 1 | 0.027 | Topic 27 | -0.026 | Topic 41 | -0.025 |
|  | (0.043) |  | (0.036) |  | (0.045) |
| Topic 12 | 0.250^**^ | Topic 29 | -0.087^*^ | Topic 42 | 0.308^***^ |
|  | (0.080) |  | (0.037) |  | (0.043) |
| Topic 13 | 0.090^*^ | Topic 30 | -0.009 | Topic 44 | 0.088^*^ |
|  | (0.038) |  | (0.044) |  | (0.039) |
| Topic 14 | -0.040 | Topic 31 | 0.670^***^ | Topic 46 | 0.945^***^ |
|  | (0.032) |  | (0.052) |  | (0.047) |
| Topic 15 | 0.117^**^ | Topic 32 | 0.063 | Topic 47 | 0.147^**^ |
|  | (0.036) |  | (0.045) |  | (0.052) |
| Topic 16 | 0.096^*^ | Topic 34 | 0.087^*^ | Topic 48 | 0.039 |
|  | (0.047) |  | (0.038) |  | (0.031) |
| Topic 2 | 0.060 | Topic 35 | -0.050 | Topic 5 | -0.031 |
|  | (0.034) |  | (0.037) |  | (0.032) |
| Topic 20 | -0.055 | Topic 36 | -0.007 | Topic 50 | -0.130^***^ |
|  | (0.036) |  | (0.041) |  | (0.028) |
| Topic 21 | 0.021 | Topic 37 | 0.163^***^ | Topic 7 | -0.425^***^ |
|  | (0.062) |  | (0.038) |  | (0.034) |
| Topic 22 | 0.092^*^ | Topic 38 | 0.888^***^ | Topic 8 | 0.358^***^ |
|  | (0.040) |  | (0.029) |  | (0.049) |
| Topic 23 | 0.035 | Topic 4 | 0.145^*^ | Topic 9 | -0.137^***^ |
|  | (0.034) |  | (0.064) |  | (0.038) |
| Topic 25 | -0.528^***^ |  |  |  |  |
|  | (0.060) |  |  |  |  |
| AIC | 66842.12 |  |  |  |  |
| BIC | 67130.84 |  |  |  |  |
| Log Likelihood | -33382.06 |  |  |  |  |
| Num. obs. | 12162 |  |  |  |  |
| Random intercept variance | 15.79 |  |  |  |  |
| Residual variance | 1.66 |  |  |  |  |
|  |  |  |  |  |  |


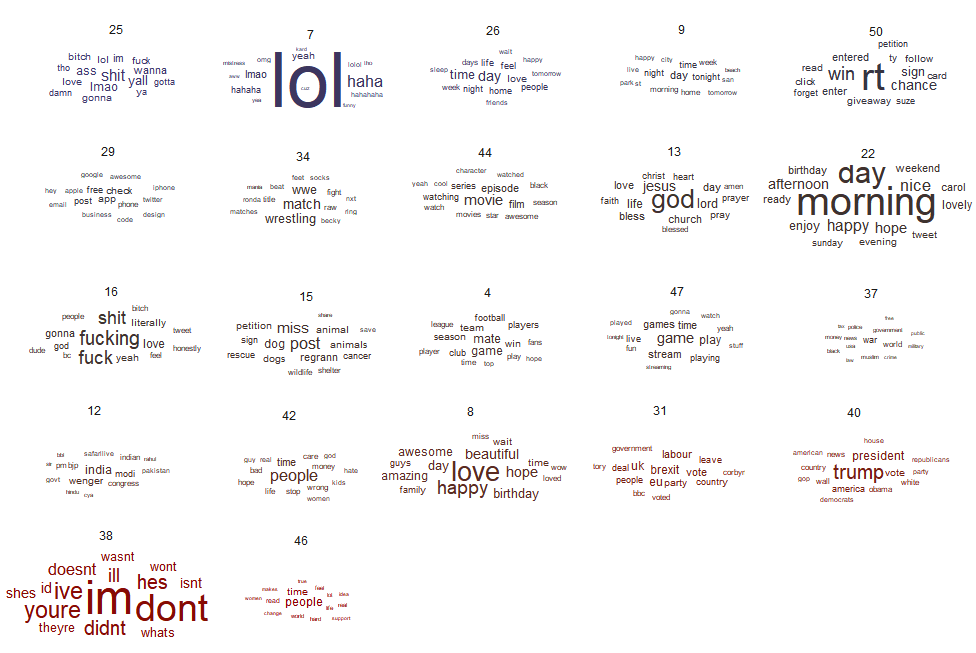


**S3 Fig. Wordclouds for the 15 top terms of the 22 topics that were significantly related to age.** The larger a term, the higher the term probability given a topic. Topics with terms in red are related to older age, topics with terms in blue are related to young age.

**S4 Table. The words removed from the NRC lexicon with their correlation with LIWC scores.** These words come from NRC but they were also found in the political topics and had negative correlations with the relevant LIWC scores (for the words in positive NRC wordlist, a correlation with LIWC positive affect scores; for the words in negative NRC wordlist, a correlation with LIWC negative affect scores).

| **Word** | ***r*** | ***p*** |  | **Word** | ***r*** | ***p*** |  | **Word** | ***r*** | ***p*** |  | **Word** | ***r*** | ***p*** |
| --- | --- | --- | --- | --- | --- | --- | --- | --- | --- | --- | --- | --- | --- | --- |
| **Positive affect** |  |  |  | **Positive affect** |  |  |  | **Positive affect** |  |  |  | **Positive affect** |  |  |
| accountable | -0.04 | .000 |  | director | -0.02 | .006 |  | main | -0.04 | .000 |  | savings | -0.04 | .000 |
| action | -0.04 | .000 |  | educated | -0.04 | .000 |  | major | -0.04 | .000 |  | scientist | -0.03 | .003 |
| afford | -0.02 | .009 |  | effective | -0.03 | .001 |  | majority | -0.10 | .000 |  | sense | -0.06 | .000 |
| aid | -0.03 | .002 |  | elect | -0.04 | .000 |  | management | -0.03 | .000 |  | sex | -0.07 | .000 |
| amnesty | -0.03 | .000 |  | electric | -0.03 | .000 |  | marriage | -0.02 | .035 |  | shoulder | -0.02 | .007 |
| apologize | -0.03 | .000 |  | electricity | -0.03 | .003 |  | mayor | -0.04 | .000 |  | soldier | -0.03 | .001 |
| armed | -0.06 | .000 |  | enforce | -0.04 | .000 |  | medical | -0.06 | .000 |  | solution | -0.03 | .001 |
| assets | -0.05 | .000 |  | equality | -0.03 | .001 |  | moderate | -0.03 | .001 |  | speech | -0.05 | .000 |
| assistance | -0.04 | .000 |  | equally | -0.02 | .026 |  | monetary | -0.02 | .028 |  | stable | -0.03 | .000 |
| assured | -0.02 | .028 |  | equity | -0.02 | .038 |  | money | -0.12 | .000 |  | statement | -0.06 | .000 |
| attention | -0.06 | .000 |  | ethics | -0.04 | .000 |  | moral | -0.05 | .000 |  | status | -0.03 | .001 |
| attorney | -0.07 | .000 |  | exchange | -0.03 | .000 |  | negotiate | -0.06 | .000 |  | store | -0.02 | .009 |
| authority | -0.04 | .000 |  | existence | -0.04 | .000 |  | oath | -0.06 | .000 |  | strategic | -0.02 | .015 |
| balance | -0.04 | .000 |  | expect | -0.07 | .000 |  | objective | -0.03 | .002 |  | study | -0.05 | .000 |
| ballot | -0.02 | .008 |  | expert | -0.04 | .000 |  | obvious | -0.06 | .000 |  | supplies | -0.02 | .034 |
| brains | -0.05 | .000 |  | explain | -0.07 | .000 |  | occupation | -0.03 | .003 |  | supply | -0.04 | .000 |
| build | -0.04 | .000 |  | extend | -0.02 | .029 |  | officer | -0.03 | .005 |  | supreme | -0.04 | .000 |
| building | -0.05 | .000 |  | extra | -0.03 | .005 |  | option | -0.05 | .000 |  | teacher | -0.02 | .021 |
| cabinet | -0.05 | .000 |  | fairly | -0.02 | .020 |  | organization | -0.04 | .000 |  | technology | -0.03 | .000 |
| candidate | -0.07 | .000 |  | food | -0.03 | .002 |  | organized | -0.02 | .015 |  | tower | -0.04 | .000 |
| capitalist | -0.06 | .000 |  | found | -0.02 | .037 |  | ownership | -0.03 | .000 |  | unity | -0.02 | .009 |
| cash | -0.03 | .001 |  | gain | -0.04 | .000 |  | pacific | -0.02 | .020 |  | university | -0.03 | .002 |
| center | -0.02 | .040 |  | govern | -0.05 | .000 |  | pardon | -0.03 | .004 |  | vaccine | -0.05 | .000 |
| chairman | -0.02 | .011 |  | green | -0.02 | .016 |  | pay | -0.14 | .000 |  | veteran | -0.02 | .048 |
| child | -0.06 | .000 |  | growth | -0.03 | .000 |  | pilot | -0.02 | .012 |  | vote | -0.03 | .000 |
| citizen | -0.05 | .000 |  | guard | -0.03 | .000 |  | planning | -0.03 | .001 |  | wages | -0.05 | .000 |
| civil | -0.08 | .000 |  | guardian | -0.03 | .001 |  | police | -0.10 | .000 |  | white | -0.13 | .000 |
| civility | -0.02 | .034 |  | hire | -0.03 | .001 |  | pope | -0.02 | .012 |  | word | -0.04 | .000 |
| clean | -0.04 | .000 |  | humanity | -0.04 | .000 |  | population | -0.08 | .000 |  | **Negative affect** | | |
| clearance | -0.03 | .002 |  | include | -0.04 | .000 |  | prepared | -0.02 | .009 |  | airs | -0.02 | .044 |
| clown | -0.04 | .000 |  | including | -0.05 | .000 |  | president | -0.09 | .000 |  | ancient | -0.02 | .025 |
| coalition | -0.06 | .000 |  | income | -0.07 | .000 |  | primary | -0.04 | .000 |  | bound | -0.04 | .000 |
| committed | -0.08 | .000 |  | increase | -0.04 | .000 |  | prime | -0.05 | .000 |  | buffet | -0.02 | .044 |
| comrade | -0.02 | .023 |  | independence | -0.03 | .002 |  | professor | -0.05 | .000 |  | cold | -0.02 | .034 |
| confirmed | -0.04 | .000 |  | influence | -0.03 | .000 |  | progressive | -0.05 | .000 |  | competition | -0.05 | .000 |
| conscience | -0.03 | .003 |  | information | -0.06 | .000 |  | protect | -0.06 | .000 |  | cross | -0.04 | .000 |
| constitutional | -0.08 | .000 |  | innocent | -0.03 | .003 |  | prove | -0.06 | .000 |  | fee | -0.02 | .038 |
| continue | -0.04 | .000 |  | inquiry | -0.03 | .001 |  | provide | -0.04 | .000 |  | hail | -0.02 | .043 |
| corporation | -0.06 | .000 |  | integrity | -0.03 | .000 |  | public | -0.12 | .000 |  | hidden | -0.04 | .000 |
| council | -0.04 | .000 |  | intellectual | -0.04 | .000 |  | pull | -0.06 | .000 |  | infamous | -0.02 | .032 |
| counsel | -0.02 | .007 |  | investigate | -0.07 | .000 |  | qualified | -0.03 | .003 |  | john | -0.06 | .000 |
| credibility | -0.05 | .000 |  | journalist | -0.06 | .000 |  | question | -0.06 | .000 |  | lemon | -0.03 | .003 |
| credible | -0.03 | .000 |  | judicial | -0.04 | .000 |  | real | -0.06 | .000 |  | lord | -0.05 | .000 |
| culture | -0.08 | .000 |  | justice | -0.06 | .000 |  | reason | -0.10 | .000 |  | mar | -0.03 | .004 |
| customer | -0.02 | .018 |  | knowledge | -0.03 | .002 |  | reform | -0.03 | .000 |  | parade | -0.02 | .026 |
| deal | -0.09 | .000 |  | labor | -0.03 | .004 |  | remains | -0.05 | .000 |  | rail | -0.02 | .031 |
| debate | -0.06 | .000 |  | land | -0.04 | .000 |  | reporter | -0.05 | .000 |  | ram | -0.02 | .040 |
| decency | -0.03 | .001 |  | lead | -0.04 | .000 |  | reproductive | -0.03 | .004 |  | spent | -0.02 | .043 |
| defending | -0.03 | .001 |  | leader | -0.06 | .000 |  | resources | -0.05 | .000 |  | stone | -0.02 | .010 |
| deliberate | -0.03 | .000 |  | legal | -0.10 | .000 |  | responsible | -0.07 | .000 |  | storm | -0.05 | .000 |
| delivery | -0.03 | .001 |  | legalized | -0.03 | .002 |  | revolutionary | -0.04 | .000 |  | wait | -0.05 | .000 |
| democracy | -0.08 | .000 |  | liberal | -0.09 | .000 |  | rising | -0.02 | .039 |  |  |  |  |
| dependent | -0.03 | .003 |  | liberation | -0.02 | .007 |  | salary | -0.04 | .000 |  |  |  |  |

### **Additional analysis of the context of value words**

To understand the context in which words representing values and civil liberties are used, we ran additional analyses inspired by collocation analysis (1,2). In this analysis, one looks at words overlapping in use with selected words. For, example, it has been shown that although Democrats and Republicans in the US use similar moral terms when discussing political topics, they use them in different ways (1). Specifically, although these groups use words related to loyalty to a similar degree, Democrats use them to draw attention to the needs of underprivileged groups (e.g., “women”, “paid leave”), whereas Republicans use these words in the context of national security or religion.

Here, we focus on words from the NRC positive word list that negatively correlated with LIWC positive scores (presented in S4 Table) that represent or are related to values, morality, or civil rights. Specifically, we select ten of these words that have the largest negative correlation with LIWC. These words were: “democracy”, “culture”, “responsible”, “protect”, “justice”, “oath”, “moral”, “credibility”, “ethics”, “humanity”. Next, we selected tweets that included one (or more) of the selected value-related words. Again, the stopwords were removed from the analysis using tidytext lists (3). We next calculated word frequencies in these tweets, separately for each identified value-related word, and selected the top ten associated words; S5 Table summarizes this analysis. Our hypothesis was that on Twitter, the words associated with values are used in the context of suggesting threat to these values rather than simply expressing them.

The results seem to be in line with this hypothesis. For example, the top word related to “credibility” is “lost” (i.e., as “lost credibility”). Furthermore, one of the top words associated with “responsible” is ”held”/”hold” and the top words associated with “oath” are “lied”/”lying”. The word “democracy” is used in the context of Brexit to a large extent and also when in association with “trump”, i.e., the President Trump. The top words associated with “ethics” are e.g., “investigation”, “committee”, “recuse”, suggesting judicial or criminal context. The top word associated with “humanity” is “crime” but somewhat contrasting are the next top words which are most likely used in a religious context, e.g., “faith”, “god”. Other words used in a religious context to a certain degree are “morality” and “protect” but the political component here is also strong (e.g., “trump”, “laws”, “country”). The word “protect” is also associated with benevolent groups such as “children”. Additionally, it seems that word “trump” occurs in the majority of the top words, which is mostly associated with controversies around President Trump and his visible presence on Twitter. Another very frequently used term in the current context is “people”.

**S5 Table. Top 10 words associated with the words from positive NRC wordlist that correlated negatively with LIWC positive scores.** Only 10 words representing values or civil rights were chosen for this analysis. The words were selected based on the correlation coefficient (starting from most negative coefficient).

| **Value word** | **Top words** | **N** | **Value word** | **Top words** | **N** |
| --- | --- | --- | --- | --- | --- |
| **democracy** | people | 511 | **culture** | people | 464 |
| **3973** | vote | 477 | **3966** | pop | 215 |
|  | trump | 302 |  | black | 180 |
|  | country | 251 |  | white | 171 |
|  | eu | 184 |  | love | 169 |
|  | voted | 169 |  | time | 169 |
|  | party | 163 |  | women | 124 |
|  | leave | 161 |  | american | 117 |
|  | uk | 152 |  | change | 116 |
|  | time | 144 |  | history | 107 |
| **responsible** | people | 274 | **protect** | people | 496 |
| **2900** | trump | 192 | **4522** | trump | 272 |
|  | held | 132 |  | country | 217 |
|  | person | 103 |  | children | 181 |
|  | government | 100 |  | laws | 179 |
|  | time | 99 |  | god | 177 |
|  | hold | 97 |  | time | 177 |
|  | actions | 81 |  | school | 176 |
|  | children | 81 |  | child | 172 |
|  | life | 76 |  | government | 165 |
| **justice** | people | 400 | **oath** | office | 135 |
| **5553** | system | 361 | **813** | trump | 91 |
|  | trump | 305 |  | constitution | 85 |
|  | social | 286 |  | people | 73 |
|  | league | 244 |  | president | 67 |
|  | court | 234 |  | investigation | 62 |
|  | time | 207 |  | lied | 61 |
|  | hope | 201 |  | lying | 49 |
|  | criminal | 190 |  | defend | 47 |
|  | department | 164 |  | protect | 46 |
| **moral** | people | 220 | **credibility** | lost | 127 |
| **1943** | compass | 203 | **975** | trump | 111 |
|  | ground | 159 |  | people | 62 |
|  | story | 154 |  | lose | 47 |
|  | trump | 117 |  | time | 45 |
|  | support | 90 |  | news | 43 |
|  | time | 63 |  | media | 38 |
|  | god | 59 |  | absolutely | 33 |
|  | values | 56 |  | party | 33 |
|  | country | 54 |  | left | 32 |
| **ethics** | trump | 68 | **humanity** | people | 234 |
| **830** | morals | 63 | **2167** | crimes | 158 |
|  | investigation | 50 |  | faith | 133 |
|  | people | 50 |  | love | 118 |
|  | committee | 44 |  | world | 112 |
|  | vote | 40 |  | time | 102 |
|  | recuse | 37 |  | trump | 99 |
|  | review | 35 |  | hope | 98 |
|  | house | 33 |  | human | 85 |
|  | barr | 30 |  | god | 61 |

Next, we split the sample into people below vs. above 50 years old. This is because our analyses seem to show inconsistency between NRC and LIWC starting at around that age. Moreover, previous research shows that people 50 years old and older are responsible for the vast majority of the political content on Twitter (4). In line with these findings, for all but one of the analyzed value-related words, people above (vs. below) 50 years of age used them more often. For example, the word “democracy” was used 996 times by people below 50., and 2,977 by people above 50. However, one exception was “culture”: here younger group used this word more as compared to the older group (2,637 vs. 1,329). Thus, we analyze the top words associated with “culture” separately for people below vs. above 50 years old in S6 Table. This analysis shows that young people use the word “culture” predominantly in the context of “pop culture” (also “love”) but also race (“black”, “white”) and gender relations (“women”, “rape”), whereas people 50+ use it predominantly in the context of world and domestic politics (“country”, “american”).

**S6 Table. Top 10 words associated with the word “culture” for people below 50 and those 50 years old or older.**

| **Word** | **People below 50 years old** | **Word** | **People 50 yeard old or older** |
| --- | --- | --- | --- |
| **culture** | **2637** | **culture** | **1329** |
| people | 297 | people | 167 |
| pop | 166 | white | 71 |
| black | 145 | country | 64 |
| time | 106 | time | 63 |
| white | 100 | change | 57 |
| love | 121 | world | 55 |
| women | 85 | american | 53 |
| history | 73 | pop | 49 |
| day | 72 | love | 48 |
| rape | 69 | life | 41 |

## Supporting information references

1. Sterling J, Jost JT. Moral discourse in the Twitterverse: Effects of ideology and political sophistication on language use among U.S. citizens and members of Congress. J Lang Polit. 2018 May 7;17(2):195–221.

2. Baker P, Gabrielatos C, KhosraviNik M, Krzyżanowski M, McEnery T, Wodak R. A useful methodological synergy? Combining critical discourse analysis and corpus linguistics to examine discourses of refugees and asylum seekers in the UK press: Discourse Soc [Internet]. 2008 May 1 [cited 2022 Jul 25]; Available from: https://journals.sagepub.com/doi/abs/10.1177/0957926508088962?journalCode=dasa

3. Silge J, Robinson D. tidytext: Text Mining and Analysis Using Tidy Data Principles in R. J Open Source Softw. 2016 Jul 11;1(3):37.

4. Bestvaver S, Shah S, Rivero G, Smith A. Politics on Twitter: One-Third of Tweets From U.S. Adults Are Political [Internet]. Pew Research Center - U.S. Politics & Policy. 2022 [cited 2022 Jul 18]. Available from: https://www.pewresearch.org/politics/2022/06/16/politics-on-twitter-one-third-of-tweets-from-u-s-adults-are-political/
